# Leukodystrophy with Macrocephaly, Refractory Epilepsy, and Severe Hyponatremia—The Neonatal Type of Alexander Disease

**DOI:** 10.3390/genes15030350

**Published:** 2024-03-11

**Authors:** Justyna Paprocka, Magdalena Nowak, Magdalena Machnikowska-Sokołowska, Karolina Rutkowska, Rafał Płoski

**Affiliations:** 1 Department of Pediatric Neurology, Medical University of Silesia, 40-055 Katowice, Poland; nmagdalena533@gmail.com; 2 Department of Diagnostic Imaging, Radiology and Nuclear Medicine, Medical University of Silesia, 40-055 Katowice, Poland; magdamas@onet.pl; 3 Department of Medical Genetics, Medical University of Warsaw, 02-106 Warsaw, Poland; karolina.rutkowska@wum.edu.pl (K.R.); rploski@wp.pl (R.P.)

**Keywords:** Alexander disease, neonatal type, refractory epilepsy, leukodystrophy, severe hyponatremia

## Abstract

Introduction: Alexander disease (AxD) is a rare neurodegenerative condition that represents the group of leukodystrophies. The disease is caused by *GFAP* mutation. Symptoms usually occur in the infantile age with macrocephaly, developmental deterioration, progressive quadriparesis, and seizures as the most characteristic features. In this case report, we provide a detailed clinical description of the neonatal type of AxD. Method: Next-Generation Sequencing (NGS), including a panel of 49 genes related to Early Infantile Epileptic Encephalopathy (EIEE), was carried out, and then Whole Exome Sequencing (WES) was performed on the proband’s DNA extracted from blood. Case description: In the first weeks of life, the child presented with signs of increased intracranial pressure, which led to ventriculoperitoneal shunt implementation. Recurrent focal-onset motor seizures with secondary generalization occurred despite phenobarbital treatment. Therapy was modified with multiple anti-seizure medications. In MRI contrast-enhanced lesions in basal ganglia, midbrain and cortico-spinal tracts were observed. During the diagnostic process, GLUT-1 deficiency, lysosomal storage disorders, organic acidurias, and fatty acid oxidation defects were excluded. The NGS panel of EIEE revealed no abnormalities. In WES analysis, *GFAP* missense heterozygous variant NM_002055.5: c.1187C>T, p.(Thr396Ile) was detected, confirming the diagnosis of AxD. Conclusion: AxD should be considered in the differential diagnosis in all neonates with progressive, intractable seizures accompanied by macrocephaly.

## 1. Introduction

Alexander disease (AxD) is a rare neurodegenerative disorder that represents a group of leukodystrophies [1,2,3]. A heterogeneous group of leukodystrophies consists of a progressive genetic disease where the white matter of the central nervous system (CNS) is predominantly affected with or without changes in the peripheral nervous system and abnormal development of the myelin sheath [3,4,5]. According to van der Knaap’s pathogenic classification of leukodystrophies, Alexander disease belongs to a group of astrocytophaties [6].

The disease is caused by mutations, usually missense alterations, in the *GFAP* gene located in chromosome 17q21 [2,5]. The gene encodes the fibrillar acid glial protein, the major intermediate filament, which takes part in the morphology and motility of astrocytes and the interaction between astrocytes and oligodendrocytes [1,7,8]. AxD inherits in an autosomal dominant manner. *GFAP* mutation results in gain-of-function mechanisms [1]. The overexpression of the mutant GFAP protein affects filament accumulation in the astrocyte cytoplasm, called “Rosenthal fibers” (RFs). RFs consist of small heat shock proteins and alpha B-crystallin. Histopathological identification of RFs confirmed AxD diagnosis before the era of genetic testing. The pathological process involves the white matter of frontal lobes and less extensively temporal lobes [5,7,9,10].

Traditionally, three types of AxD were distinguished, divided by the age of diagnosis: infantile, juvenile, and adult type [1,11]. For the predominant infantile form, initial symptoms occur before the age of 2 years. These symptoms involve seizures, ataxia, hyperreflexia, megalencephaly, feeding difficulties, and spasticity. The juvenile form is revealed in school-age children with features such as bulbar and pseudobulbar signs (dysphasia, dysarthria), ataxia, spasticity with possible epilepsy, and intellectual disability. Patients with this form of AxD are normocephalic, and the progression of the disease is milder than in previous types, with surveillance rates ranging from early adolescence to 30 years [1,9]. Adult form, much less frequent than other types, is characterized by variable clinical presentation with progressive ataxia, quadriparesis, eye movement abnormalities, palatal myoclonus, dysautonomia, and postural defects. Neuroradiological findings typically involve the brainstem and cervical spinal cord. The survival is also diverse since patients may live from a few years to decades after their first symptoms [11,12,13]. Recently, the neonatal form was distinguished from infantile due to a more severe clinical course and a very unfavorable prognosis [11,14].

The disease usually begins at the infantile age. However, the age of onset ranges from the first weeks of life to adulthood and is based on the type of the disease [3,9,14]. Usually, the early onset of the disease predisposes to a more severe course with increased mortality [1]. Macrocephaly, developmental deterioration, progressive quadriparesis, and seizures are the most characteristic features of AxD. However, the symptoms may differ according to the type of the disease. For infantile onset, the involvement of cerebral white matter is typical and contrary to adult onset, which marks the brainstem involvement [1,3,11,14].

The diagnosis is made based on typical clinical presentation and characteristic changes in neuroimaging studies [3]. To establish diagnosis based on MRI, four of five criteria should be met: extensive cerebral white matter changes with frontal predominance, a periventricular rim with high signal on T1-weighted images and low signal on T2-weighted images, abnormalities of basal ganglia and thalami, brain stem abnormalities, and contrast enhancement of particular gray and white matter structures [10]. Due to variable clinical presentation with the atypical course of AxD, the diagnosis should be confirmed by molecular genetic testing [1,11,12]. Treatment of AxD is only symptomatic, focusing on seizure control, improving motor dysfunction, and management of hydrocephalus with a ventriculoperitoneal shunt implementation [2].

## 2. Materials and Methods

Suspecting genetic etiology, Next-Generation Sequencing (NGS) of a panel with 49 genes related to Early Infantile Epileptic Encephalopathy (EIEE) was performed. Analyzed genes included *ALDH7A1*, *ALG13*, *ARX*, *CACNA1A*, *CASK*, *CDKL5*, *CHD2*, *DNM1*, *FOXG1*, *GABRA1*, *GABRB3*, *GABRG2*, *GNAO1*, *GRIN1*, *GRIN2A*, *GRIN2B*, *HCN1*, *HNRNPU*, *IQSEC2*, *KCNA2*, *KCNB1*, *KCNQ2*, *KCNQ3*, *KCNT1*, *MBD5*, *MECP2*, *MEF2C*, *PCDH19*, *PNPO*, *POLG*, *PRRT2*, *SCN1A*, *SCN1B*, *SCN2A*, *SCN8A*, *SCN9A*, *SIK1*, *SLC25A22*, *SLC2A1*, *SLC35A2*, *SLC9A6*, *SPTAN1*, *STXBP1*, *SYNGAP1*, *TBC1D24*, *TBL1XR1*, *TCF4*, *UBE3A*, and *ZEB2.*

Then, exome sequencing was performed on the proband’s DNA extracted from peripheral blood using Twist Human Core Exome 2.0 + Comp Spike-in + Twist mtDNA Panel (Twist Bioscience, South San Francisco, CA, USA) according to manufacturer’s instructions. The enriched library was paired-end sequenced (2 × 100 bp) on NovaSeq 6000 (Illumina, San Diego, CA, USA). The mean coverage of the sample was 173x; 99.7% of the target was covered ≥20x and 99.8% of the target was covered ≥10x. Data analysis and variants’ prioritization were performed using an in-house bioinformatic pipeline as previously described [15]. Segregation of the prioritized variant in the proband’s family members was analyzed using amplicon deep sequencing (ADS). We studied the proband’s sample, along with his healthy parents and sister, using ADS with a Nextera XT Kit (Illumina, San Diego, CA, USA) and paired-end sequencing.

MRI (magnetic resonance imaging) of the brain was performed using Artiste 1.5 T GEM (General Electric, GE Healthcare, Milwaukee, WI, USA). Imaging protocol included standard sequences with contrast enhancement in control examinations. The data were transferred to a commercially available Workstation ADW 3.2.

## 3. Case Description

A 6-week-old infant was admitted to the Pediatric Neurology Department due to drug-resistant epilepsy. The child was born in the 38th week of an uncomplicated pregnancy with an Apgar score of 10 points, a bodyweight of 3220 g, and a head circumference of 35 cm (frontal fontanelle 1 × 1 cm). The patient’s family history was unremarkable.

**Figure 1:**
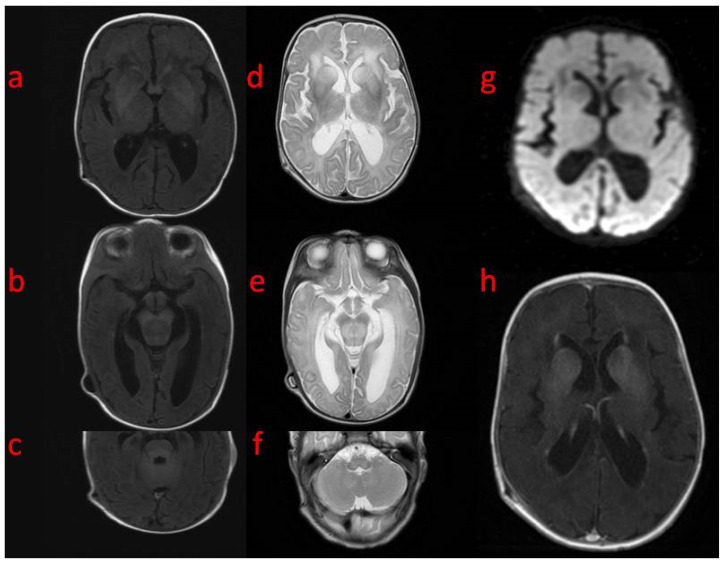
Brain MRI (**a**–**c**) (5th week of life): axial T1 on the level of (**a**) basal ganglia, (**b**) midbrain, (**c**) pons with superior cerebellar peduncles with an abnormal signal in the frontal white matter, basal ganglia without signal changes in the pons and cerebellum (**d**–**f**): axial T2—(**d**) significantly increased frontal white matter and basal ganglia signal, enlarged caudate nuclei, (**e**) increased signal and volume in the midbrain, (**f**) increased signal in the oblongate medulla; (**g**) axial DWI—generalized even signal without differentiation except for increased diffusivity in the frontal white matter; (**h**) axial T1 post-contrast: enhancement of basal ganglia.

**Figure 2:**
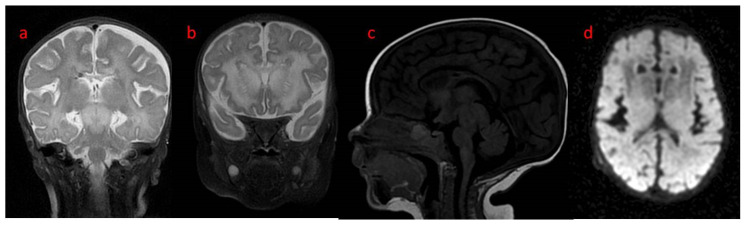
Brain MRI control after 1 week: (**a**,**b**) coronal T2: more generalized white matter T2 hyperintensity with a low signal rim around ventricles, marked symmetrical perivascular spaces in internal capsules; (**c**) sagittal T1 with an abnormal signal in cortico-spinal tracts in the brain stem; (**d**) axial DWI: slightly better signal differentiation with generally abnormal diffusivity.

In the first weeks of life, the child presented with increasing apathy, severely limited spontaneous activity, and a lack of eye tracking. At the age of 5 weeks, a physical examination revealed the bulging frontal fontanelle and separation of the cranial sutures. A transfontanelle ultrasound examination showed a significantly enlarged supratentorial ventricular system with a ventricular index of 0.57 (N: 0.2–0.25). Signs of increased intracranial pressure led to ventriculoperitoneal shunt implantation. The surgery resulted in the resolution of the dehiscence of the sutures with limited improvement of the neurological state. MRI of the brain performed after neurosurgery revealed decreased T1 and increased T2 white matter signals in both hemispheres with frontal domination, midbrain, cortico-spinal tracts, and enlarged basal ganglia with heterogenous signals and enhancement. DWI (diffusion-weighted imaging) showed generalized abnormality and an even signal with increased diffusivity in the frontal white matter (Figure 1). In a 1-week follow-up, white matter signal changes were generalized, and symmetrical periventricular spaces in internal capsules were shown (Figure 2).

Focal-onset motor seizures with secondary generalization led to the introduction of phenobarbitals. After 3 days, due to recurrent cluster seizures, antiepileptic treatment was modified with levetiracetam.

The elementary laboratory test results (inflammation parameters, ionogram, liver and kidney parameters, lactic acid, and ammonia) were within the normal range. The CSF analysis revealed significantly reduced glucose concentration (24 mg/dL, 22 mg/dL) and glucose index (0.2) with pleocytosis of 130/μL. The normal cerebrospinal fluid (CSF) culture led to the exclusion of infection of the valvular system. PCR analysis of pathogens related to meningitis was also negative. Control CSF analysis revealed the normalization of cytosis and a glucose index of 0.36. Suspecting the glucose transporter type 1 (GLUT-1) deficiency syndrome, a ketogenic diet was introduced. Therapy resulted in slight improvement as the patient could occasionally move his limb and open his eyes spontaneously. However, the patient hardly achieved full ketosis despite the introduction of the proportion in the 3:1 ketogenic diet.

EEG examination showed slow theta waves with single sharp waves. There were no abnormalities in the ultrasound of the abdominal cavity. In echocardiography, collateral vessels from the descending aorta and patent foramen ovale (PFO) without hemodynamically significant structural heart disease were found. Ophthalmological examination revealed optic nerve discs with blurred outlines, arched slightly above the level of the fundus.

Based on the diagnostic work-up, lysosomal storage disorders, organic acidurias, aminoacidopathies, and fatty acid oxidation defects were excluded. The epilepsy panel by Next-Generation Sequencing (NGS) showed no abnormalities. GLUT-1 deficiency was also excluded.

The re-intensification of epileptic seizures was observed—clonic seizures of focal onset, mainly right-sided, and myoclonus of variable location with correlating EEG changes occurred. Treatment was modified temporarily with phenytoin and then clobazam, after which a reduction in focal seizures was observed, and then carbamazepine was added. Despite multidrug treatment, tonic seizures with saturation decreases appeared, leading to apnea and consciousness disturbances. The patient was transferred to the Intensive Care Unit, and after stabilization, the antiepileptic therapy was continued.

During the follow-up, at the age of 4 months, the patient presented with a head circumference of 43 cm, a frontal fontanelle of 3 × 3 cm, and a lack of visual and verbal contact. Neurological examination revealed axial hypotonia with peripheral hypertonia, symmetrical tendon reflexes in the upper limbs, and polyclonic in the lower limbs with bilateral foot clonus. Anti-seizure treatment in polytherapy with an increased dose of carbamazepine and a ketogenic diet was continued. The control EEG showed an abnormal burst-suppression activity (SBA) with localized, lateralized, and generalized lesions. During hospitalization, massive fluctuations in sodium concentrations were observed (the lowest values of 113 mmol/L), which were corrected with intravenous (2.2% and 3% NaCl) and oral supplementation (10% NaCl). After an endocrinological consultation, fludrocortisone was implemented. Due to high CRP (99.4 mg/L) values, broad-spectrum antibiotic therapy was used, and potential sources of infection were excluded. Control CSF analysis showed increased protein concentration (589 mg/dL) with normal glucose concentration (39 mg/dL) and cytosis (5/μL).

In proband’s sample missense heterozygous variant in the *GFAP* gene (NM_002055.5:c.1187C>T, p.(Thr396Ile)) involving the alteration of a conserved nucleotide (PhyloP100 7.34) was identified. The studied variant was excluded in the proband’s parents and sister, suggesting it was caused by a de novo event (germinal mosaicism cannot be excluded). The variant was absent in the gnomAD control database (v4; https://gnomad.broadinstitute.org, accessed on 19 February 2024) and in the in-house database of > 12,000 WES of Polish individuals. In silico BayesDel_addAF, BayesDel_noAF, FATHMM_MKL, and PrimateAI tools predict a pathogenic outcome for this variant. According to the American College of Medical Genetics and Genomics (ACMG), the variant was predicted as “likely pathogenic” (strong, PS2 strong, PM1 moderate, PM2 supporting).

The c.1187C>T variant was previously described in a patient with Alexander disease [14]. According to the OMIM (Online Mendelian Inheritance in Man; https://omim.org/about, accessed on 19 February 2024 ) database, pathogenic variants of the *GFAP* gene (including mainly missense variants) lead to Alexander disease in the autosomal dominant mode of inheritance (MIM#203450).

**Figure 3:**
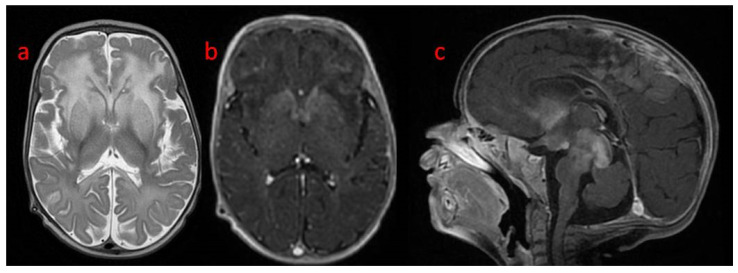
Brain MR at the 1st follow-up (4th month of life): (**a**) axial T2 pronounced progression of frontal white matter hyperintensity; post-contrast T1 (**b**) axial and (**c**) sagittal: enhancement of basal ganglia and along cortico-spinal tracts.

**Figure 4:**
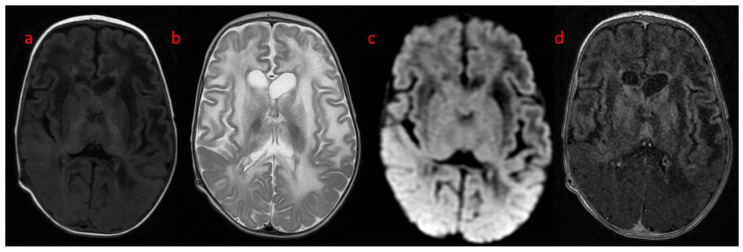
Brain MR at the 2nd follow-up (4th month of life): axial (**a**) T1, (**b**) T2, (**c**) DWI, (**d**) post-contrast T1: progression of signal abnormality, fluid collections at the frontal horns level.

A follow-up brain MRI showed the progression of the abnormal, diffused, increased signal of white matter with frontal domination. Moreover, periventricular narrow bands of high T1 and low T2 signals, inhomogeneous signals from the basal nuclei with the most intense changes in the heads of the caudate nuclei, lenticular nuclei, and anteromedial parts of the thalamus, were observed. The examination also revealed post-contrast enhancement of subcortical nuclei and along the cortico-spinal tracts (Figure 3 and Figure 4).

**Figure 5:**
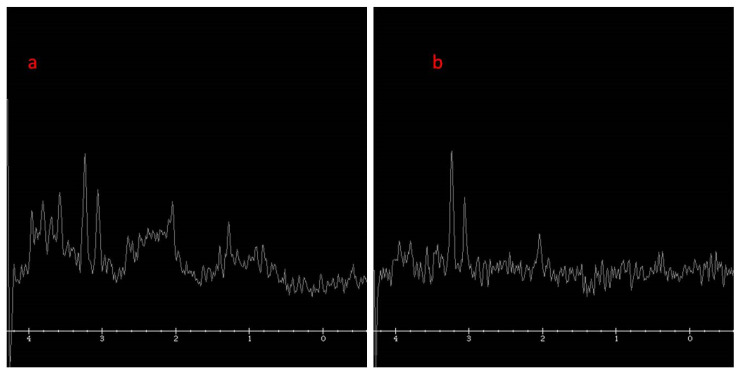
MRS (magnetic resonance spectroscopy): (**a**) TE 35; (**b**) TE 144.

Magnetic resonance spectroscopy (MRS) examination revealed decreased NAA at both times and reversed the Cho/Cre ratio. Slightly elevated lac, glucose, and inositol peaks were observed (Figure 5). The patient died at the age of 4 months.

## 4. Discussion

Leukodystrophies are a heterogeneous group of progressive neurological disorders with variable symptoms [4,15]. Apart from this variability, this group of hereditary disorders has a common feature: the underlying pathological process primarily and mostly affects the white matter of CNS, independent of peripheral nervous system involvement [4,15]. In recent years, due to the progress of molecular genetic testing, which has become more accessible, the number of known leukodystrophies has significantly enlarged [4]. This variety contributes to the challenge in the clinical pathway for establishing the diagnosis of leukodystrophies. 

Patients with Alexander Disease present variable clinical presentations and prognosis, with non-obvious phenotype–genotype correlation [11,14,16]. Therefore, in AxD, not only genetic tests but also the age of disease onset and clinical features, including findings in brain MRI, are crucial in the diagnostic process and prognosis. This explains the rapid development of new clinical classifications of AxD in recent years, which sometimes may be confusing and need explanation [4,16]. 

Apart from the traditional classification described before, two revisited and corresponding classifications have been provided by Prust et al. [1] and Yoshida et al. [17], and both distinguish the main two types of AxD. More severe, type I by Prust et al. and resealing cerebral form by Yoshida et al. are characterized by early onset, usually before the fourth year of life, seizure, macrocephaly, and paroxysmal deterioration with psychomotor developmental delay. In this subtype, classic radiological features include the involvement of supratentorial structures in MRI [1,12,17]. In contrast, milder type II and corresponding bulbospinal and intermediate types present across the lifespan with a predominant clinical picture as bulbar symptoms, ocular movement abnormalities, and palatal myoclonus, but unlike type I, developmental deficits are not characteristics and atypical radiological features in MRI, like white matter, affected mainly in the posterior fossa and abnormalities or atrophy of the medulla oblongata and cervical spinal cord [1,17].

Both these classifications lacked a neonatal type distinguished from variable infantile AxD, which was proposed by Springer et al. [11]. This separation is necessary due to a uniform pattern of severe clinical presentation in neonatal AxD [10,11,14,16,18,19]. To date, the neonatal form was described only in approximately 20 patients [14,16,19]. Therefore, we provided a detailed description of the neonatal type of AxD with rapid progression to expand the clinical spectrum of AxD.

As presented in our patient, for the neonatal form, two predominant features are obligatory: macrocephaly/hydrocephalus with raised intracranial pressure and often drug-resistant seizures. The seizures may be multiform and frequent, typically occurring in the first weeks of life, even as a first symptom. Hydrocephalus is caused by pathological astroglia proliferation, leading to aqueductal stenosis. Moreover, due to the early, severe progression of the disease, patients characterize a lack of developmental progression with the absence of postural acquisitions. Other typical symptoms include hypotonia, vomiting, and failure to thrive [10,11,16,18,19]. Unlike the infantile type, prominent spasticity, ataxia, or upper motor symptoms are not observed in neonatal AxD. The prognosis is very unfavorable. The first symptoms occur at a mean age of 25 days, ranging from birth to 4 months. Rapid progression leads to death within the first months of life, and survival to the second year of life is rare. In some cases, increased cerebrospinal fluid (CSF) protein concentration may be observed [10,16]. Diagnosis should be confirmed by neuroradiological examination. Typically, radiological features include severe white matter abnormalities with fronto-temporal predominance, the involvement of basal ganglia, and periventricular enhancement [10,11,18,19]. Furthermore, radiological features of neonate AxD may be detected in the fetus during prenatal neuroimaging [20,21]. Notably, the latest AxD classification separates patients into five subgroups, Ia, Ib, Ic, Id, and II, where subgroup Ia corresponds with the neonatal type with three checkpoints: developmental delay, no postural acquisitions, and early fatal course [14].

The identified variant was previously described in a patient presenting with a cerebral/neonatal form of AxD. The patient’s phenotype was classified as type Ia of AxD. The patient had a lack of postural acquisition and a rapidly progressive course, leading to death within the eighth month of life (PMID:34865968), suggesting a deleterious effect of the c.1187C>T variant. Given the similarities in the clinical course of both patients, the c.1187C>T variant is responsible for the same particularly severe course of type 1 Alexander disease, leading to death in just the first months of life. Due to limited clinical data of the previously described patient, we expand the phenotype of the c.1187C>T variant presenting a rapid course of refractory epilepsy and severe hyponatremia.

Our patient presented with chronic hyponatremia, which is not a typical feature for AxD. Due to the normal cortisol range, adrenal insufficiency was excluded. The filtration function of the kidneys was preserved, although analysis of tubular transport indicated renal sodium loss, as fractional sodium excretion in the state of profound hyponatremia was 1.5%, indicating a significant impairment of the ability to reabsorb sodium. This disorder may be the result of a direct defect of the renal tubules or incomplete compensatory action of hormonal mechanisms, including aldosterone. Another possible explanation is cerebral salt-wasting syndrome. In the described patient, low glucose concentration in CSF together with pleocytosis firstly required the exclusion of infection as the most common cause. After the normalization of cytosis, isolated low CSF glucose accompanied by drug-resistant epilepsy led to the suspicion of GLUT-1 deficiency. In this clinical situation, genetic tests should confirm diagnosis, as intractable seizures are often the first manifestation of this metabolic disturbance [22]. However, child genetics tests and poor response to the ketogenic diet excluded GLUT-1 deficiency. Leen et al. revealed that isolated low CSF glucose may occur in patients with ventriculoperitoneal shunts without CNS infection [23], which may be a possible explanation for our patient disturbances.

Even though the latest classifications include the neonatal type of AxD, making the diagnosis itself may be challenging due to the need to exclude many other disease entities. Macrocephaly is a common symptom of demyelinating dystrophies; therefore, Canavan disease, megalencephalicleukoencephalopathy with subcortical cysts (MLCs), and L2HGA should be taken into account in the differential diagnosis [4,15].

Moreover, we suggest that due to possible nonspecific clinical course in the differential diagnosis of AxD, other neurometabolic diseases should be excluded, such as MLD, GM1, Sandhoff disease, Tay–Sachs disease, CLN1, CLN2, Krabbe disease, and mannosidosis, in our patient. The white matter and basal ganglia abnormalities may also mimic mitochondrial disorders. Therefore, in neonatal AxD, extensive neurometabolic workup is necessary to enable an effective diagnosis pathway. Moreover, as seizures often become the first symptom of neonatal AxD, other etiologies of neonatal seizure should be excluded as infectious, brain malformation genetic epilepsy, and acute symptomatic seizures [24,25]. Notably, when suspecting leukodystrophies, neuroimaging axial and sagittal T1W, axial T2W, and FLAIR should be ordered and expanded with post-contrast T1W [4].

The variable course of AxD results in a lack of clear phenotype–genotype correlation [14,16]. Even though neonatal AxD distinguishes itself from other types by more established clinical courses, few cases have been described. Expanding the clinical and genetic spectrum of neonatal AxD is necessary to identify future therapy targets and enable the design of new treatment options, especially as trials are being set up to test antisense nucleotides in patients with Alexander disease [15].

## 5. Conclusions

Establishing the diagnosis of neonatal AxD may be challenging due to the rapid course of the disease. However, this condition should be considered in all neonates with macrocephaly with hydrocephalus, accompanied by progressing, intractable seizures. In those cases, diagnostic work-up needs to be expanded to brain MRI and MRS with necessary follow-up examinations and neurometabolic tests. Moreover, precisely describing the second patient with the c.1187C>T variant, we suggest that this variant is associated with the same severe clinical course of neonate AxD, leading to death within the first months of life.

## Data Availability

Data are contained within the article.
